# Almost Everything We Need to Better Serve Children of the Opioid Crisis We Learned in the 80s and 90s

**DOI:** 10.3389/fpubh.2018.00289

**Published:** 2018-10-16

**Authors:** Kimberly A. Horn, Robert P. Pack, Robert Trestman, Gerard Lawson

**Affiliations:** ^1^Virginia Tech-Carilion Research Institute, Roanoke, VA, United States; ^2^College of Public Health, East Tennessee State University, Johnson City, TN, United States; ^3^East Tennessee State University, College of Public Health, Johnson City, TN, United States; ^4^East Tennessee State University (ETSU), Center for Prescription Drug Abuse Prevention and Treatment, Johnson City, TN, United States; ^5^Virginia Tech School of Medicine, Roanoke, VA, United States; ^6^Carilion Clinic, Roanoke, VA, United States

**Keywords:** children of drug abusers, opioid prevention, drug abuse prevention, children of addicts, child welfare

## Abstract

Opioid use disorder impedes dependent parents' abilities to care for their children. In turn, children may languish in unpredictability and persistent chaos. Societal responses to these children are often guided by a belief that unless the drug dependent parent receives treatment, there is little help for the child. While a preponderance of the drug dependence research is adult-centric, a significant body of research demonstrates the importance of not only addressing the immediate well being of the children of drug dependent caregivers but preventing the continuing cycle of drug dependence. The present commentary demonstrates through a brief review of the US history of drug dependence crises and research from the 1980s and 1990s, a range of “tried and true” family, school, and community interventions centered on children. We already know that these children are at high risk of maladjustment and early onset of drug dependence; early intervention is critical; multiple risk factors are likely to occur simultaneously; comprehensive strategies are optimal; and multiple risk-focused strategies are most protective. Where we need now to turn our efforts is on how to effectively implement and disseminate best practices, many of which we learned in the 1980s and 1990s. The greatest opportunity in both changing the nature of the opioid epidemic at scale and influencing rapid translation of existing research findings into policy and practice is not in asking what to do, but in asking how to do the right things well, and quickly.

## Introduction

The United States has a long history of drug dependence dating back to its origin, including several opioid epidemics in the last two centuries (1, 2). While it is distinct in some ways, today's opioid epidemic is an echo of past illicit drug crises in that children and youth are vulnerable. In the current epidemic, babies are often born on opioids, resulting from their mother's opioid dependence. Anguished cries and difficulty sleeping and feeding/nursing in the crucial first days of life mark their own withdrawal, termed Neonatal Abstinence Syndrome (NAS) (3). In a study of 28 states, the incidence of NAS increased almost 300% from 1999–2013, rising from 1.5 to 6.0 cases per 1000 hospital births. But, the current situation in some states is much worse. For example, the incidence of NAS in WV is 51 per 1000 live births; at least two counties in WV reported more than 100 cases per 1000 live births (3).

Children of parents with opioid use disorder (OUD), when discovered, are often placed with grandparents, other family members, or enter the foster-care system. Children remain in the homes of their drug dependent parents or caregivers and are at great risk for neglect, abuse, or worse.(4, 5) In the 1970's opioid epidemic, we learned that many depressed and anxious children resort to silence, upholding a fundamental family rule: no one talks about the problem, not to each other, and especially not to outsiders (4). Living in the context of this perceived imperative, a majority of these children exist in emotional incarceration where they are unable to reach out to others for support (5, 6). It is not surprising that we don't grasp the magnitude and number of affected children. As stated by Carol Levine, a MacArthur Fellow and renowned expert on the traumatic impact of epidemics on children, “No one knows how many of these vulnerable children there are in the US because no one is counting. They remain hidden in families with addiction until a crisis erupts and law enforcement or child welfare agencies get involved” (7). Such family dynamics hold true in the current opioid epidemic.

Societal aid for affected children is often guided by a belief that unless the drug dependent parent receives treatment, there is little help for the child. This view is debilitating because most opioid users never seek treatment. In turn, an entire family system remains frozen in uncertainty until the addicted individual accepts treatment, is forced into treatment, becomes justice-involved, is hospitalized, or dies. To understand how we arrived at this place, it is important to revisit the history of drug dependence in the US.

The US is no stranger to drug epidemics (8, 9). In the 1970s it was heroin and hallucinogens. The 1980s drug crisis shifted to cocaine and crack, and in the 1990s crystal methamphetamine was the drug of focus. Today, despite comprising < 5% of the world's population, the US consumes over 80% of the global opioid supply (10) with 5% experiencing dependence (10). Historically, the US response to drug crises prioritizes policy, legal action and criminalization, adult treatment, and relapse prevention. These actions are manifest in the 1956 Narcotic Control Act, the Controlled Substances Act of 1970, the formation of the DEA as a part of our “War on Drugs” in 1973; and the first Tobacco Industry Lawsuit in 1988 (11). As drug dependence continued to increase in the 1970s, and throughout the 1980s and 1990s, research on the causes and consequences of dependence also increased. The National Institute of Drug Abuse (NIDA) formed in 1974 and became a formal part of National Institutes of Health (NIH) in 1992 (9). The federal government also created the Alcohol, Drug Abuse, and Mental Health Agency (ADAMHA; now the Substance Abuse and Mental Health Services Administration SAMHSA) in 1992 to increase availability of resources for prevention and treatment of substance use and mental health disorders (12).

While a preponderance of the drug dependence research is adult-centric, a significant body of research demonstrates the importance of not only addressing the immediate well being of the children of drug dependent caregivers but preventing the continuing cycle of drug dependence. In fact, as touted by NIDA, by the late 1980s scientists were well on their way to building “… the scientific base for effective drug dependence prevention programs by identifying individual, family, school, and neighborhood factors that place children at risk for drug dependence. Researchers have since developed a broad array of effective family, school, and community programs that target these factors” (9).

## Discussion

### What we already know about children of drug dependent parents

#### Children are at high risk of maladjustment and early onset of drug dependence

These children are likely to develop affective, behavioral, cognitive, and interpersonal adverse childhood experiences (ACEs) that manifest at home, school, and in peer groups (13). See examples summarized in Table 1. Children of opioid dependent parents or caregivers experience diminished levels of self-esteem and use isolation or withdrawal to cope (4, 14, 15). Moreover, they tend to normalize the dysfunction in their homes, and display poor interpersonal and social skills, low social attachment, and favorable attitudes toward drug dependence (16–18). Evidence from previous opioid crises tells us that these children are at risk for a variety of psychiatric disorders, including eventual drug dependence (17, 19, 20). Recent findings from longitudinal studies on ACEs underscore the profound impact of parental drug dependence on children's behavior and mental health (21).

**Table 1:** Examples multi-level risk factors (13).

**INDIVIDUAL FACTORS**
Exposure to opioids in utero
Low birth weight
Failure to thrive
Early antisocial behaviors (e.g., lying and impulsivity)
Low self-worth and low self esteem
Depression
Delinquency and history of legal problems
Rejection of adult values
Low social bonding
**FAMILY**
Parental rejection and inadequate nurturing
Low bonding and attachment from birth
Parental permissiveness
Inadequate supervision and neglect
Inconsistencies in parental discipline and parenting practices
Family separation or lack of family closeness
Poor family communication
Family conflict (persistent)
Family history of alcohol or drug dependence
Child physical abuse and sexual abuse
Poverty and low family income
**SCHOOL**
Poor school readiness
Early adjustment difficulties
Conduct disorder
Impulsivity and aggression at school
Poor interpersonal relationships at school
Low commitment to and poor academic performance
Low bonding and attachment to school
Low involvement in extracurricular activities
Poor attendance, truancy, or dropping out of school
**PEER**
Susceptibility to peer pressure
Friends who have favorable attitudes toward or use alcohol, tobacco, or other drugs
Rejection by peers
Aggression toward peers (persistent)
Victim of bullying

#### Early intervention is critical

Early detection and treatment is important to minimize the potential for enduring functional impairment and suffering (17, 19, 20). Childhood development research on resiliency (22, 23) shows interventions that reduce exposure to risk and adversity will: (1) improve competencies, personal resources, and coping skills; (2) prevent or reduce early onset of maladaptive behavior; and (3) target specific groups who show a higher probability of developing high risk behaviors than the general population (24). Applicable today, early intervention with children of parents with OUD can moderate their responses to known risk factors, eliminating or buffering later risk factors (22, 23).

#### Multiple risk factors are likely

The presence of one risk factor or stressor occurring in isolation may not increase the likelihood that a child develops adjustment problems (25). But, when two or more stressors co-occur, the chances are two to four times greater that the child will develop significant problems (25). Fitting within this framework of cumulative risks, effective prevention for these children requires a focus on multiple risk factors as it is unlikely that children of individuals with OUD experience to risk factors singularly (26, 27) Refer to Table 1.

#### Comprehensive strategies are optimal

Many drug dependence prevention programs for children occur in general settings where large groups of youth congregate (e.g., school classrooms). We know that these universal programs will be minimally effective for children affected by parental OUD because they: (1) are didactic; (2) assume comparable levels of risk for all children; and (3) do not address psychological, behavioral, cognitive, and interpersonal risk factors characteristic of children of drug dependent parents (28). Information-focused sessions presume that children make decisions about health or risky behavior in a values-expectancy framework (i.e., if a child values health then they will engage in healthy behavior). This approach ignores the complexity of the social, physiological, developmental, and external realities of their lives. Additionally, universal programs typically give little consideration to other factors such as cultural and social perspectives, socioeconomic levels, or specific sub-populations (16, 29, 30). This assumption does not suggest that prevention should preclude the use of broad prevention interventions. Researchers and practitioners must follow the evidence showing the importance of selective or indicated interventions for children affected by OUD consisting of appropriate content, intensity, and dosage (26, 31). The most effective interventions will target the biological, psychological, or social risk factors.

#### Multiple risk-focused strategies are most protective

Comprehensive “integrated delivery systems, long-term continuity of services, coupling of child development and family services, parent education, and inclusion of prenatal care….” increase the chances of positive cumulative impact (p.7) (31, 32). A notion of a cumulative strategies approach for children of drug dependent parents aligns with selected or indicated interventions. More specifically, interventions should consider the co-occurring delivery of at least two of five basic prevention strategies (27) inclusive of affective information, social and life skills training, alternative play activities, academic support networks, and healthy lifestyle mentoring. A comprehensive approach for children affected by parental OUD suggests that effective prevention strategies will: (1) focus on younger children; (2) offer specific risk-focused rather than generalized interventions; (3) address a range of behavioral, psychological, and cognitive risk factors that are common to children from families coping with OUD, and (4) consist of multiple components that target two or more risk factors together (27).

Selective interventions developed and tested in the 1980s, 1990s, and into the 2000s show evidence in support of approaches designed to decrease risk and increase protective factors involving one or domains of individual, peer, family, school, and community (16). Several such models exist in the literature: The Family Management Model of Adolescent Substance Abuse (14); The Intensive Family Prevention Services Model (33); The Social Ecological Model of Adolescent Substance Abuse (34); Communities that Care (35); Life Skills (36, 37); Strengthening Families (34); Focus on Kids (38); Multisystemic Therapy (39), and PROSPER (40), to name a few. Many of these evidence-based programs appear in repositories such as the SAMHSA Evidence-Based Practice Resource Center (URL: https://www.samhsa.gov/ebp-resource-center), Social Programs that Work (URL: http://evidencebasedprograms.org), and the Coalition for Evidence-based Policy (URL: http://toptierevidence.org). Bearing utility for practitioners, including pediatricians and family medicine physicians working with children of the current opioid crisis, these websites provide information in lay-person language appropriate for local—or regional level tailoring and implementation, including key outcomes, effectiveness across a variety of settings, and costs for implementation.

As with the epidemics of the 1980s and 1990s, schools are the primary place where children receive prevention services (41). Evidence shows that school counselors can have integral roles in the lives of children of drug dependent parents. Important for children affected by parental OUD, professional school counselors are better trained to identify and intervene than they were in the previous century, in large part due to the standardization of training through accreditation standards by the Council on the Accreditation of Counseling and Related Educational Programs (CACREP, URL: http://www.cacrep.org/wp-content/uploads/2018/05/2016-Standards-with-Glossary-5.3.2018.pdf). CACREP standards require training in “the theories and etiology of addictions and addictive behaviors” and “evidence-based counseling strategies and techniques for prevention and intervention.”

### What we don't know and how the gaps can inform rapid results research

Where we are falling short is understanding of how to implement and disseminate our best practices, many of which we learned in the 1980s and 1990s. Perhaps the greatest opportunity in both changing the nature of the opioid epidemic at scale and influencing rapid translation of research into policy and practice is not in asking *what* to do, but in asking *how* to do the right things *well*, and quickly. There are many effective tools to fight the epidemic as explained by Mathis et al. in a study outlining effective approaches all along the continuum of OUD (42). It is clear that effective prevention programming pays the most dividends for return on investment, with as many as $15 saved for every dollar spent on primary prevention for drug dependence (43). Part of the challenge is in how we compel people to adopt these tools. Since children are typically in school, school-based programming is an obvious starting point.

Several key questions should be considered. First, how do we persuade educational institutions to prioritize evidence-based prevention programming delivered by trained facilitators when federal and state pressures on school systems are largely focused on academics and not health? Second, how do we make large scale prevention programs sustainable after grant funding ends? Primary prevention programming is often subject to the vagaries of grant funding. Unfortunately, grants do not always go to the places that need them the most. For prevention programming to be sustainable, the stakeholders must find clear value in the programming, and there must be funding mechanisms to support it (44). Third, how do we tailor interventions to be scalable and efficient? Effective primary prevention programming is ideally theory-based, made up of myriad skill-developing activities with peers, and with a high expectation that the facilitator will deliver the intervention as intended (i.e., fidelity) (45). Fourth, how does adoption of excellent prevention programming become routinized over creation and evaluation of new programs, which is costly, time consuming and uncertain on efficacy? Entire academic careers can focus on crafting novel and effective interventions. But these same interventions, despite the strength of evidence of effectiveness, are not disseminated to the populations that need them the most. Rotheram-Borus and colleagues evoke a disruption in intervention dissemination, calling for simpler and better programs that are more market-oriented and responsive to the public's needs than current practices (46). Finally, how will NAS-affected children experience development and what will their experience be in school? An emerging literature on this topic indicates that NAS-affected school-age children present with significant problems that will affect their development and academic progress, and will cause significant deficits in learning and knowledge by the time they enter high school (47). Further, the areas of the country most affected by the long-standing prescription drug abuse epidemic that now includes other opiates like heroin and fentanyl, are already under-resourced for children that may need individualized education plans. These issues also beg for the engagement of pediatricians and family medicine providers in prevention interventions as early as possible. For instance, providers can receive training to provide screening and brief intervention among their patients, particularly adolescent and young adult female patients, as reproductive-age women are increasingly a focal point of the opioid crisis (10). Trained providers are also in positions to detect NAS symptoms when they may have been otherwise missed. At a minimum, pediatricians and family medicine providers have opportunities unique other providers to jointly educate children and families about OUD, including safe storage practices. All of these primary and secondary prevention efforts have demonstrated efficacy (13).

Having knowledge about the effectiveness of these programs or interventions does not mean that we shouldn't seek new knowledge. Of course, we should. But we should also focus on the best methods to improve the Reach, Adoption, Effectiveness, Implementation, and Maintenance (REAIM) of evidence-based practices and approaches (48). This simple but powerful tool, outlining key tenets of implementation science, can help guide our work and improve health outcomes for locally and nationally (49).

In conclusion, as evidenced by the rich citations in this commentary, deliberately cited from the 1980s and 1990s, there is much to gain by seeking knowledge from the archives and putting it to work for these children now. We simply don't have time to reinvent the wheel. In fact, there is not a moment to lose in this grand challenge.

## Author contributions

KA conceived the topic of focus and wrote significant portions of the manuscript. RP, RT, and GL contributed in writing to sections of the manuscript. All authors provided final review and approval of the full manuscript.

### Conflict of interest statement

The authors declare that the research was conducted in the absence of any commercial or financial relationships that could be construed as a potential conflict of interest.
